# Polysaccharide Peptide Extract From *Coriolus versicolor* Increased T_max_ of Tamoxifen and Maintained Biochemical Serum Parameters, With No Change in the Metabolism of Tamoxifen in the Rat

**DOI:** 10.3389/fphar.2022.857864

**Published:** 2022-04-01

**Authors:** Valentina Razmovski-Naumovski, Benjamin Kimble, Daunia Laurenti, Srinivas Nammi, Hisayoshi Norimoto, Kelvin Chan

**Affiliations:** ^1^ NICM Health Research Institute, Western Sydney University, Penrith, NSW, Australia; ^2^ School of Science, Western Sydney University, Penrith, NSW, Australia; ^3^ South West Sydney Clinical Campuses, Discipline of Medicine, University of New South Wales Sydney, Sydney, NSW, Australia; ^4^ School of Medicine, Western Sydney University, Penrith, NSW, Australia; ^5^ R&D Centre of PuraPharm Corporation Ltd. and PuraPharm (Nanning) Pharmaceutical Co. Ltd., Hong Kong, Hong Kong SAR, China; ^6^ School of Pharmacy and Biomolecular Sciences, Liverpool John Moores University, Liverpool, United Kingdom

**Keywords:** polysaccharide peptide, traditional Chinese medicine, yunzhi, cancer, drug interaction, pharmacokinetics

## Abstract

**Background:** Polysaccharide peptide (PSP) extract of *Coriolus versicolor* (L.) Quél. (1886) (*Trametes*; Polyporaceae) is increasingly used in cancer to support the immune system. However, its interaction with tamoxifen is unknown.

**Aim of the study:** To investigate the effect of a PSP extract on the pharmacokinetics, biochemical parameters, and depletion of tamoxifen.

**Methods:** The pharmacokinetic and biochemical parameters of tamoxifen (20 mg/mL oral single dose and repeated dosing for 12 days) was investigated in female Sprague Dawley rats with or without PSP (340 mg/kg orally for 7 days) (*n* = 5 per group). Tamoxifen (5 µM) depletion rate with PSP (10–100 μg/mL) was measured in female rat hepatic microsomes *in vitro*.

**Results:** Compared to tamoxifen alone, the time to reach maximum concentration (T_max_) significantly increased by 228% (4.15 ± 1.15 versus 13.6 ± 2.71 h) in the single tamoxifen dose with PSP and 93% (6 ± 2.17 versus 11.6 ± 0.4 h) in the repeated tamoxifen dosing with PSP (*p* < 0.05). No significant changes in the area-under-curve and maximum concentration were observed in the single dose and repeated tamoxifen dosing plus PSP compared to tamoxifen alone. Pharmacodynamically, the repeated tamoxifen dosing with PSP maintained 19 out of 23 hepatic, renal and cardiac biochemical serum parameters in rats compared to untreated rats (*p* > 0.05). PSP extract did not significantly alter *in vitro* intrinsic clearance of tamoxifen compared to tamoxifen control.

**Conclusion:** With the increased use of PSP as an adjunct therapy, this study highlights the importance of clinician’s knowledge of its interaction with tamoxifen to avoid compromising clinical actions and enhancing clinical therapy.

## Introduction

In recent times, people with cancer have turned to natural products to enhance recovery from anticancer therapy and its deleterious effects on the immune system (Boon and Wong, 2004; Cassileth and Vickers, 2005; Meijerman et al., 2006; Oneschuk and Younus, 2008; Zhang et al., 2018). *Coriolus versicolor* (L.) Quél. (1886) (*Trametes*; Polyporaceae) or “Yunzhi” in Chinese, is an edible, colourful mushroom with prized energising and healing effects, and has been used in folk medicine, especially traditional Chinese medicine, to treat herpes, liver disorders including hepatitis, chronic fatigue syndrome, upper respiratory, urinary and digestive tract infections, strengthen physique and increase energy (Chu et al., 2002; Cruz et al., 2016). Much of the immune-stimulating properties of *C. versicolor* have been attributed to proteoglycans in the form of polysaccharide peptide (PSP) (Hor et al., 2011; Saleh et al., 2017) and polysaccharide krestin (PSK) (Habtemariam, 2020). Studies have suggested that these proteoglycans in *C. versicolor* may be able to fight cancer and boost the immune system, reduce the toxic effects and pain of chemotherapy and radiation therapy; increase the effectiveness of chemotherapy; prolong and improve the quality of life of people with cancer (Ng, 1998; Kidd, 2000; Chu et al., 2002; Cui and Chisti, 2003). A systematic review and meta-analysis has shown overall survival benefits in breast, gastric and colorectal cancer chemotherapy when combined with *C. versicolor* (Wong et al., 2012; Zhong et al., 2019). In Japan, *C. versicolor* is approved as an immunotherapeutic agent for cancer. Thus, it is important to understand potential interactions between natural products such as *C. versicolor* and anticancer drugs as interactions can undermine the anticancer therapy and/or illicit toxic responses to the body (McCune et al., 2004; Meijerman et al., 2006; Seely and Oneschuk, 2008; Yeung et al., 2018).

The nonsteroidal, antineoplastic agent tamoxifen is a first line drug in the prevention and treatment of oestrogen receptor-positive breast cancer which make up around 70% of breast cancers (Drago et al., 2019). Its extensive use in clinical practice makes it an important drug to examine potential interactions. In previous rat studies, tamoxifen has shown enhanced bioavailability with other natural products such as quercetin (Shin et al., 2006), silybinin (Kim et al., 2010), curcumin (Cho et al., 2012), baicalin (Li et al., 2011) and naringin (Choi and Kang, 2008) potentially due to the inhibition of cytochrome P34A (CYP3A4). These results show that combining tamoxifen with natural compounds may have clinical therapeutic implications. Although clinical evidence suggests that *C. versicolor* is beneficial as an adjunct treatment in breast cancer (Wong et al., 2005; Standish et al., 2008; Saleh et al., 2017), there is little information on whether or how it interacts with tamoxifen (Cheng et al., 2018).

To provide guidance for clinical co-therapy, the aim of this study was to examine the pharmacokinetics, biochemical interaction and depletion of tamoxifen with a commercial *C. versicolor* PSP extract using a female Sprague-Dawley rat model. The rat animal model was chosen as the metabolic disposition of tamoxifen in the rat resembles that in humans which will help to further extrapolate these results to clinical practice (Lien et al., 1991).

## Materials and Methods

### Materials and Reagents

Tamoxifen citrate salt, internal standard verapamil hydrochloride, potassium dihydrogen phosphate, magnesium chloride and ortho-phosphoric acid (H_3_PO_4_) were purchased from Sigma-Aldrich (St. Louis, MO, United States). Isoflurane was purchased from Cenvet (Kings Park, WA, Australia) while gavage needles (18G) were purchased from Livingstone, NSW, Australia. An 18G needle was used in these studies because it is thinner than 16G and can easily pass through the oesophagus without causing any damage to mucosal membrane and distress to the animal. The high-performance liquid chromatography (HPLC) grade acetonitrile, methanol and hexane were from Analytical Science (Sydney, NSW, Australia). Irradiated rat pellet diet was purchased from Speciality Feeds (Glen Forrest, WA, Australia).

The commercial PSP extract (ONCO-Z^®^) was supplied by PuraPharm International (H. K.) Ltd., Hong Kong. The PSP extract is made from the fruiting body of dried 100% wild *C. versicolor* extracted in deionised water and contains absorbable peptidoglycan (APG). The standardised extract (340 mg) is equivalent to 2.83 g of dried *C. versicolor* and comes from a USP-accredited biological procedure for standardisation involving a freeze-drying process. The extract was verified by the U.S. Pharmacopoeia (The U.S. Pharmacopeia, 2019) in August 2009 and comprises of at least one peptide linked glucan, with glucose molecules as a monosugar connected by a 1→3 linkage. The crude extract (molecule weight of 0.5–40 kDa) comprises of an average 4.7% peptide/protein composition, 55% neutral sugar and 4.8% uronic acid. The purified extract has a molecular weight of 0.3–5 kDa (average 2.6 kDa) and is highly water soluble. The amino acid sequence of the protein/peptide moiety was determined to be Asp-Cys-Pro-Pro-Cys-Glu (Chow and Chu, 2006). To maintain verification status, the manufacture of the extract must continue under the same conditions. A voucher specimen was deposited at NICM Health Research Institute, Western Sydney University, Australia (Batch no: A1301475). A partial structure of the PSP polysaccharide component is proposed in a previous study (Hor et al., 2011).

### Dosing Preparations

The PSP extract was prepared in distilled water at a concentration of 170 mg/mL and administrated according to the animal weight (2 mL/kg). The concentration was chosen to reflect a high dose (340 mg/kg) and was under the considered lethal dose of more than 5,000 mg/kg (Hor et al., 2011).

Tamoxifen was prepared at a concentration of 15.2 mg/mL dissolved in water and administrated by oral gavage according to the animal’s weight (2 mL/kg). As tamoxifen is given orally to patients, oral gavage allowed the drug to be delivered directly to the stomach and this minimises the error associated with free feeding. The tamoxifen dose was chosen to keep the plasma concentrations above the detection limit (Choi and Kang, 2008).

### Animals

Adult female Sprague-Dawley rats (over 12 weeks of age; 215–340 g) were bred in-house in the School of Medicine Animal Housing Facility, Western Sydney University. This rat species, gender and methods described has been used in other tamoxifen drug interaction studies, and this information provides a basis for this *in vivo* study (Shin et al., 2006; Choi and Kang, 2008; Kim et al., 2010; Kim et al., 2020). Throughout the experiment, three rats were weight-matched and randomly housed in the facility in Green Line individually ventilated cages (IVC) (Techniplast, United States), in a temperature-controlled room (22 ± 3°C), 50–60% relative humidity, under a 12 h light-dark cycle. The animals were acclimatised for at least 1 week with a normal diet and water *ad libitum*. During the study, rat observations included posture, coat hair, activity, movement, breathing, alertness, appetite changes/vomiting, dehydration, eyes, nose, faeces, and abnormal bleeding. This was monitored daily using an animal health monitoring sheet. Initial and final weights were recorded. The rats were anaesthesised using a high dose of isoflurane (and/or ketamine and xylazine as an option) to bring them into a surgical plane of anaesthesia and bled completely by cardiac puncture. Isoflurane anaesthesia has shown no significant effects on plasma metabolites (Davis, 1996).

### Pharmacokinetic Interaction Study of Tamoxifen

The pharmacokinetic interaction study consisted of two main parts: a single oral dose of tamoxifen and repeated dosing of tamoxifen (to reflect chronic dosing and achieve steady state), with PSP extract fed orally for 7 days in both scenarios.

Thirty rats were randomly divided into six groups of five rats each. There were two control groups: Grp 1a control (untreated, only water) and Grp 1b PSP-treated control (PSP extract administered orally daily at 340 mg/kg for 7 days), with serum collected on day 8.

There were two tamoxifen control groups: Grp 2a single tamoxifen oral dose (30.4 mg/kg tamoxifen citrate equivalent to 20 mg/kg tamoxifen base) on day 8 with serum collected on the same day; and Grp 2b repeated tamoxifen oral dosing (20 mg/kg per day) for 13 days with serum collected on day 13. In humans, the steady state for tamoxifen is achieved in about 4–6 weeks (Kisanga et al., 2004). In rats, it has been shown that steady state is reached in 3 days (Lien et al., 1991). Thus, 13 days for Grp 2b depicted the chronic administration of tamoxifen in clinical practice, and this timeframe allowed for any biochemical changes to occur.

There were two PSP treatment with tamoxifen groups: Grp 3a single tamoxifen oral dose (20 mg/kg) on day 8, pre-treated with PSP extract administered orally at 340 mg/kg daily for 7 days (to emulate pre-treatment of the natural product) with blood sampling done on day 8 to discern PSP’s effect on a single tamoxifen dose; and Grp 3b repeated tamoxifen oral dosing (20 mg/kg per day) for 12 days plus PSP extract administered orally daily at 340 mg/kg from days 5 to 11, with blood sampling done on day 12. For Grp 3b, the repeated daily dosing of tamoxifen reflects chronic administration in clinical practice, with PSP taken daily during active treatment. Tamoxifen was administered in the morning, whilst PSP extract was administered in the afternoon to provide a time gap between the two interventions.

Three rats were sampled, in the morning, at any one time. This allowed the blood sampling time frames to be achieved. The rats were fasted for at least 12 h prior to blood sampling to ascertain whether the extract continued to have any effect on the drug. During blood sampling, each rat was anesthetised by inhalation with isoflurane which allowed quicker and cleaner access for blood sampling and rapid recovery. Blood samples (up to 0.3 mL) were collected from the lateral saphenous vein at 0, 0.25, 0.5, 0.75, 1, 2, 4, 8, 12, and 24 h. Multiple small samples are unlikely to cause hypovolaemia to the animal (Diehl et al., 2001). The blood was centrifuged after sampling, and the serum samples were stored at −80°C until analysis.

All the animal experiments were carried out in accordance with the strict guidelines of the Animal Research Regulation 2005 (NSW) and the Australian code of practice for the care and use of animals for scientific purposes. Approval was endorsed by the Animal Ethics Committee of Western Sydney University [Animal Research and Teaching Proposal (ARTP) Approval Number: A9873] using the principles of replacement, reduction and refinement. The reporting of the results followed the Animal Research Reporting of *In Vivo* Experiments (ARRIVE) guideline (Kilkenny et al., 2010).

### Liquid Chromatography Conditions

Chromatography was performed with a Shimadzu LC-20AT delivery unit consisting of a DGU-20A degassing solvent delivery unit, SIL-20A auto injector, CTO-20A column oven and SPD-A detector (Kyoto, Japan). Chromatographic separation was achieved by a Synergy MAX-RP-80A (4 µ, 150 × 4.6 mm) (Phenomenex, Torrance, CA, United States) attached to a 1 mm Optic-guard C-18 pre-column (Optimize Technologies, Alpha Resources, Thornleigh, Australia) in ambient temperature. The isocratic mobile phase, using a modified method as described previously (Antunes et al., 2013; Yang et al., 2013), comprised of 50 mM potassium phosphate buffer (pH 2.15) and acetonitrile (55:45, v/v) using verapamil hydrochloride as the internal standard. The mobile phase was delivered at a flow rate of 1 mL/min. The eluent was monitored at 280 nm ultra-violet detection.

### Preparation of Stock and Working Solutions of Tamoxifen

A stock solution of tamoxifen citrate (2 mM: equivalent to 0.371 mg/mL of tamoxifen) was prepared in methanol and was further diluted with methanol to give a series of working solutions of 0.12, 0.23, 0.46, 0.93, 1.86, 3.71, and 7.42 μg/mL. A stock solution of verapamil (50 μg/mL) was prepared in methanol. Both stock solutions were stored at −20°C and working solutions were freshly prepared from the prepared stock solution when required. Standard curves were prepared by adding known concentrations of tamoxifen and verapamil to drug-free rat plasma.

### Preparation of Standard and Quality Control Samples of Tamoxifen

The LC method was validated according to the International Council for Harmonisation guideline (The International Council for Harmonisation 1996). Low, middle and high concentrations of tamoxifen samples (0.023, 0.186, and 0.742 μg/mL, respectively) were prepared by spiking 10 µL of the tamoxifen working solutions (0.23, 1.86, and 7.42 μg/mL) into 100 µL of blank rat serum and stored at −20°C. Serum calibration standards (0.012–0.742 μg/mL) were freshly prepared for each analysis by spiking 10 μL of working solutions of tamoxifen into 100 μL of blank pooled rat serum which was pre-thawed at room temperature.

### Sample Preparation

Briefly, 100 µL of serum samples were spiked with 5 µL of verapamil (final concentration: 2.5 μg/mL) after the deproteinisation with 100 µL of acetonitrile. The samples were extracted twice with n-hexane (900 µL) and the organic layer was removed and dried at 30°C for 30 min under vacuum in a Speed Vac concentrator (Thermo Scientific, United States). The dried samples were reconstituted with the mobile phase (90 µL), and 30 µL was injected into HPLC system.

### Pharmacokinetic Analysis of Tamoxifen

The pharmacokinetic parameters of tamoxifen were determined by a non-compartmental analysis using PKSolver (Zhang et al., 2010). Maximum serum concentrations (C_max_) and time to reach maximum concentration (T_max_) for both the single and repeated tamoxifen dosing experiments were determined by visual inspection of the serum concentration vs. time curve. The elimination constant rate (k_el_) was estimated by semi-log linear regression of the terminal slope, and elimination half-life (t_1/2_) was estimated by ln2/k_el_. Area under the curve (AUC) and area under the first moment curves (AUMC) from 0 to last observed concentration (AUC_0-t_ and AUMC_0-t_, respectively) were determined by the linear trapezoidal method.

### Serum Biochemical Parameters of Single and Repeated Oral Doses of Tamoxifen

For the 24 h time point of the rat’s serum, 23 biochemical serum parameters, incorporating hepatic [e.g., alkaline phosphatase (ALP), alanine aminotransferase (ALT), total protein, albumin, globulins, total bilirubin], renal (e.g., creatinine, urea, calcium, phosphate) and cardiac (e.g., cholesterol, triglycerides, bile acids) diagnostics, were examined for all six rat groups. The samples were analysed by an external veterinary diagnostic laboratory [Veterinary Science Diagnostic Service (VSDS), University of Queensland, Australia] who were blinded to the control/experimental groups.

### *In Vitro* Intrinsic Clearance of Tamoxifen Alone and in Combination With PSP Extract

The depletion of tamoxifen alone and in the presence of PSP extract was performed using female rat liver microsomes (Sigma-Aldrich, Australia) *in vitro* using methods as previously described (Yang et al., 2013). The rat model emulates the metabolism of tamoxifen in humans (Lien et al., 1991). Briefly, 5 µL of tamoxifen (0.5 mM dissolved in 25% acetonitrile) was preincubated with or without the PSP extract (10, 50, and 100 μg/mL) in 0.5 mL of 0.1 M phosphate buffer (pH 7.4) containing a nicotinamide adenine dinucleotide phosphate (NADPH) regenerating system (1 mM NADP+, 0.8 U glucose-6-phosphate dehydrogenase and 3 mM glucose-6-phosphate; Promega, Sydney, NSW, Australia) and 3 mM magnesium chloride in an open air shaking water bath at 37°C for about 3 min. Acetonitrile in the final incubation was 0.25%. After the preincubation, the enzymatic reaction was then initiated by adding 0.5 mg/mL of female rat hepatic microsomes. During the incubation, 100 µL aliquots were removed at time (t) = 0, 20, and 30 min. Each extracted aliquot was mixed with 200 µL of ice-cold acetonitrile to deactivate the enzymatic reaction. The resultant mixture was vortexed and centrifuged at 14,000 × *g* for 10 min, and the supernatant (30 µL) was directly injected to the HPLC system for analysis as described in the previous section. The overall *in vitro* intrinsic clearance (Cl_int_) was estimated by the substrate depletion method using *in vitro* t_1/2_ approach (Obach, 1999) rather than the product formation method (Rane et al., 1977) as it is known that tamoxifen undergoes multiple CYP-mediated metabolism. Briefly, using the AUC of tamoxifen at t = 0 as 100% of substrate, the AUC of the other time points were converted to a percentage of the substrate remaining, plotted as natural log of remaining drug versus incubation time, and the slope of the regression line, represented as depletion rate of constant (-k), was used for estimation of the *in vitro* t_1/2_ by the following equation:

$$in\;vitro\;{\text{t}}_{1/2}=-0.693/\text{k}$$

Subsequently, *in vitro* Cl_int_ was calculated by following formula:

$$\left(\text{0}\text{.}693/\mathrm{in}\;\mathrm{vitro}\;{\text{t}}_{1/2}\right)\text{×}\left(\text{mL}\;\text{incubation}\;\text{volume}/\text{mg}\;\text{microsomal}\;\text{protein}\right).$$

### Statistical Analysis

The data was expressed as the means ± standard error of mean (SEM) of the separate experiments using Windows Excel. The pharmacokinetic data was compared using unpaired *t*-test with two-tailed *p* value. The author (BK) who completed the pharmacokinetic data was blinded to the actual experimental groups. The biochemical parameters were compared by one-way analysis of variance (ANOVA), followed by Tukey’s/Dunnet’s method for multiple comparisons using the program GraphPad Prism (San Diego, United States). The author who performed the biochemical statistics was not blinded to the groups. *p* values less than 0.05, 0.01, and 0.001 were considered statistically significant. All pharmacokinetic parameters are expressed as mean ± standard deviation, except for the elimination t_1/2_ (harmonic mean ± pseudo-standard deviation).

## Results

### HPLC Method Validation for the Determination of Tamoxifen in Rat Serum

The retention times for both tamoxifen and verapamil were approximately at 7.9 and 2.2 min, respectively. The total run time for each sample was 16 min. The concentrations of pharmacokinetic samples were determined by non-weighted least square linear regression of the serum calibration curve prepared daily and ranged from 0.0116 to 0.742 μg/mL (*r* ^2^ ≥ 0.998), where peak area ratios of tamoxifen to verapamil were used. The calibration curve was y = 4.042x-0.015 (based on the average of three calibration curves). Based on the calibration curves, a lower limit of quantification was estimated at 0.0232 μg/mL where the precision and accuracy were within accepted criteria [< 15% of coefficient of variation (CV) and within 20% of nominal concentration, respectively]. A test for lack of fit revealed no departure from linearity for the calibration curve (F = 0.186; df = 5, 14; *p* = 0.186), indicating that the linear model was valid. For quality control (QC) samples (0.0232, 0.0927, and 0.742 μg/mL), the intra- (*n* = 3) and inter-day (*n* = 3 days) precision, expressed as CV, ranged from 2.81 to 4.69% and 8.53–14.10%, respectively. Intra-day and inter-day accuracy of QC samples were within 95.08–115.43% and 84.00–117.55%, respectively.

### Serum Concentration-Time Profile of Single Tamoxifen Oral Dose in Rats With and Without PSP Extract

**FIGURE 1:**
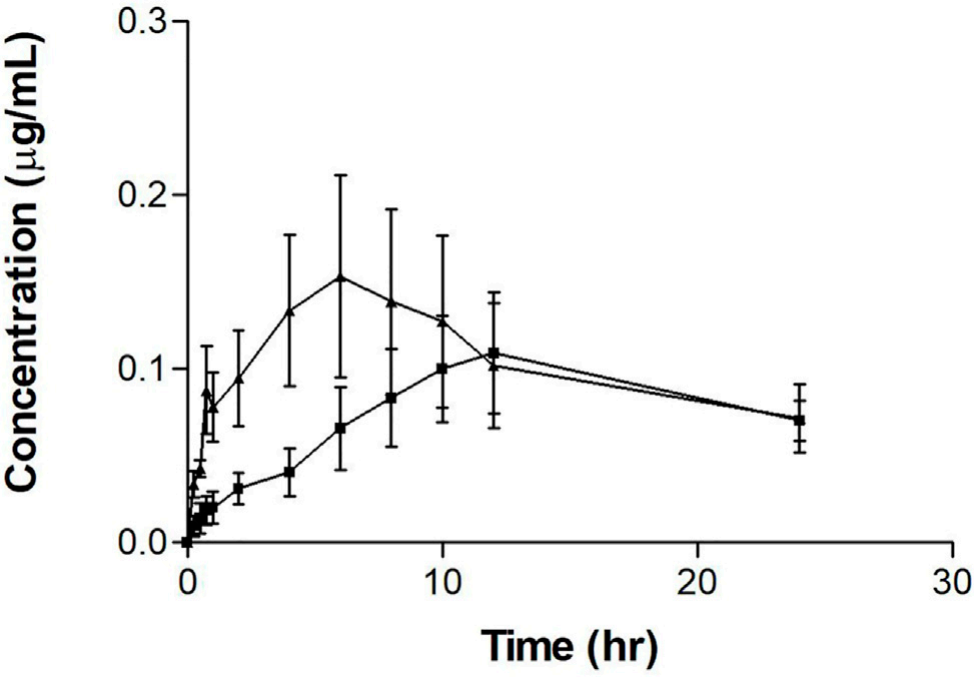
Mean serum concentration-time profiles of tamoxifen after a single oral dose of tamoxifen (20 mg/kg on day 8) (▲) with or without PSP extract (pretreatment 340 mg/kg orally for 7 days) (■) in female Sprague-Dawley rats. Each point represents as the mean ± SEM (*n* = 5). Note: timepoints at 10, 12, and 24 h are *n* = 4 for tamoxifen plus PSP extract.

The single tamoxifen oral dose serum concentration-time profile with and without PSP extract is shown in Figure 1. It is observed that the single tamoxifen oral dose plus PSP extract showed a different serum-concentration profile compared to tamoxifen alone. The curve for tamoxifen plus extract exhibits a slower rate to its peak concentration in the first 10 h compared to the steeper single tamoxifen oral dose curve. The error bars indicate greater variability in the time points for the single dose which may indicate the extract’s control on tamoxifen’s absorption.

### Effect of PSP Extract on the Pharmacokinetics of Single Tamoxifen Dose in Rats

The pharmacokinetic parameters for the single tamoxifen dose are shown in Table 1. The AUC, C_max_ and t_½_ for tamoxifen were comparable to literature values in other tamoxifen interaction studies (Shin et al., 2006; Choi and Kang, 2008; Kim et al., 2010). However, there was a significant difference between T_max_ values between the groups, with PSP extract increasing the value by three times. Although there was no significant difference between the two regimens for maximum concentration (C_max_) and area under the serum concentration-time curve from 0 to 24 h (AUC_0–24h_) (*p* > 0.05), PSP extract pretreatment showed a tendency to reduce both the C_max_ and AUC_0–24h_ of tamoxifen. PSP extract pretreatment also reduced the rate and extent of absorption of tamoxifen as seen from the decreased slope of the absorption phase, thus affecting the C_max_ and AUC_0–24h_. Limitations in sampling time points precluded further calculations of the parameters using the PKSolver software.

**TABLE 1:** Non-compartmental pharmacokinetic parameters of single oral tamoxifen dose (20 mg/kg on day 8) with/without PSP extract (340 mg/kg orally for 7 days prior) in female Sprague-Dawley rat serum. Data was expressed as mean ± SEM (*n* = 5).

Parameter (unit)	Single tamoxifen dose	Tamoxifen + PSP extract
AUC_0–24h_ (μg·h/mL)	2.41 ± 0.82	1.55 ± 0.48
T_max_ (h)	4.15 ± 1.15	13.6 ± 2.71^a^
C_max_ (μg/mL)	0.17 ± 0.05	0.11 ± 0.03
t_½_ (h)	17.7 ± 3.42	NC

a *p* < 0.05 significant difference as compared to tamoxifen. AUC_0–24 h_: area under the serum concentration-time curve from 0 to 24 h; C_max_: maximum concentration; T_max_: time to reach maximum concentration; t_½_: terminal half-life; NC = Not calculated due to limited sampling time points.

### Serum Concentration-Time Profile of Repeated Tamoxifen Oral Dosing in Rats With and Without PSP Extract

**FIGURE 2:**
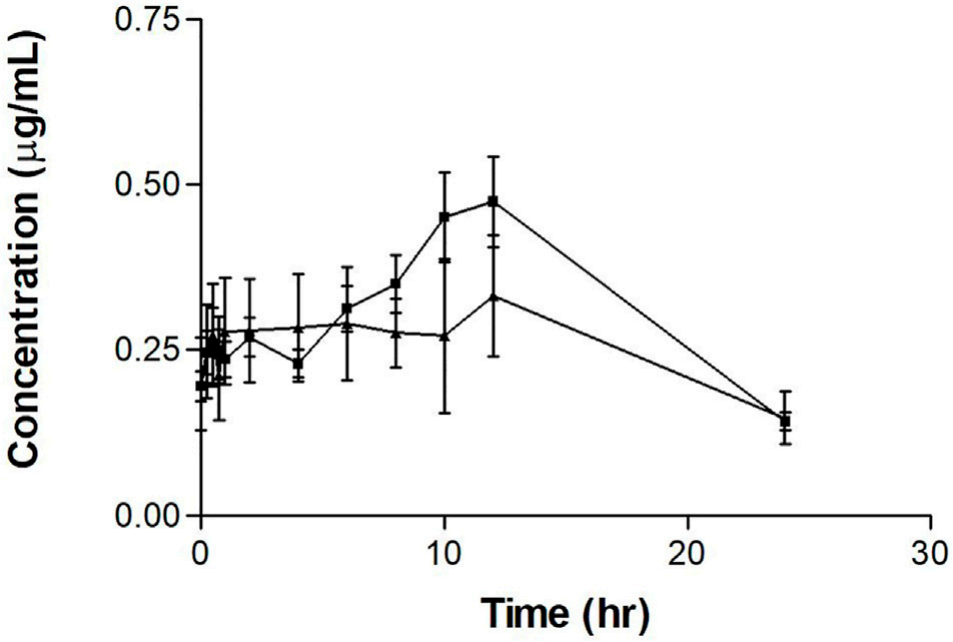
Mean serum concentration-time profiles of tamoxifen after repeated oral tamoxifen dosing (20 mg/kg for 12 days) (▲) with or without PSP extract (340 mg/kg orally on days 5–11) (■) in female Sprague-Dawley rats. Each point represents as the mean ± SEM (*n* = 5).

The repeated tamoxifen oral dosing serum concentration-time profile with and without PSP extract is shown in Figure 2. The curve for tamoxifen plus extract shows an increased peak after 10 h.

### Effect of PSP Extract on the Pharmacokinetics of Repeated Tamoxifen Oral Dosing in Rats

The tamoxifen repeated-dose pharmacokinetic parameters are shown in Table 2. As for the single dose experiments, the results showed a significant difference (*p* < 0.05) between the T_max_ values, with T_max_ almost doubling with the administration of PSP extract. There was no significant difference between the tamoxifen only and tamoxifen plus PSP extract for the other measured parameters. As a comparison, there was no significant difference between the T_max_ values of the single and multiple dose experiments for tamoxifen alone and for tamoxifen and PSP extract (*p* > 0.05).

**TABLE 2:** Non-compartmental pharmacokinetic parameters of repeated tamoxifen oral dosing (20 mg/kg for 12 days) with/without PSP extract (340 mg/kg orally on days 5–11) in female Sprague-Dawley rat serum. Data was expressed as mean ± SEM (*n* = 5).

Parameter (unit)	Repeated tamoxifen dosing	Tamoxifen + PSP extract
AUC_0–24h_ (μg·h/mL)	6.40 ± 1.51	7.63 ± 0.95
T_max_ (h)	6 ± 2.17	11.6 ± 0.4^a^
C_max_ (μg/mL)	0.40 ± 0.08	0.48 ± 0.068
t_½_ (h)	26 ± 13.7	NC

a *p* < 0.05 significant difference as compared to tamoxifen. AUC_0–24 h_: area under the serum concentration-time curve from 0 to 24 h; C_max_: maximum concentration; T_max_: time to reach maximum concentration; t_½_: terminal half-life; NC = Not calculated due to limited sampling time points.

### Serum Biochemical Parameters of Single and Repeated Tamoxifen Oral Dosing With or Without PSP Extract

The results of the biochemical rat serum parameters for control (i.e., untreated and PSP-treated), single and repeated tamoxifen oral dosing with or without PSP extract are shown in Table 3. Twenty-three parameters were examined. There were no significant differences for the PSP-treated, single tamoxifen oral dose and single tamoxifen oral dose pretreated with PSP extract compared to untreated control (*p* > 0.05) for all the parameters tested.

**TABLE 3:** Biochemical parameters of single (20 mg/kg) and repeated tamoxifen oral doses (20 mg/kg per day over 12 days) with/without PSP extract (340 mg/kg orally for 7 days) in female Sprague Dawley rat serum.

Parameter (unit)	Control (untreated)	PSP extract only	Single tamoxifen dose + PSP extract	Single tamoxifen dose	Repeated tamoxifen dosing + PSP extract	Repeated tamoxifen dosing
Na (mmol/L)	142.74 ± 0.55	144.70 ± 0.32	143.84 ± 0.96	145.84 ± 2.42	145.52 ± 1.82	148.34 ± 3.78
K (mmol/L)	5.30 ± 0.35	5.50 ± 0.14	5.50 ± 0.12	5.68 ± 0.29	5.78 ± 0.64	7.66 ± 0.61**
Mg (mmol/L)	1.04 ± 0.07	1.01 ± 0.03	1.08 ± 0.04	1.00 ± 0.02	1.07 ± 0.08	1.11 ± 0.07
Ca (mmol/L)	2.65 ± 0.07	2.67 ± 0.04	2.66 ± 0.02	2.60 ± 0.04	2.39 ± 0.09	2.25 ± 0.17*
PO_4_ (mmol/L)	2.45 ± 0.16	2.22 ± 0.14	2.54 ± 0.12	2.22 ± 0.04	2.84 ± 0.21	2.86 ± 0.22
Cl (mmol/L)	101.80 ± 0.55	103.48 ± 0.31	102.40 ± 0.65	104.64 ± 1.69	107.74 ± 0.91	112.78 ± 3.04***
CO_2_ (mmol/L)	23.94 ± 0.71	23.32 ± 1.50	20.46 ± 0.66	25.22 ± 1.16	21.14 ± 0.61	18.48 ± 1.65*
AST (U/L)	121.96 ± 20.27	163.92 ± 25.86	158.62 ± 18.85	137.34 ± 19.12	219.36 ± 57.26	401.18 ± 50.34***
ALT (U/L)	49.42 ± 6.46	44.82 ± 4.26	45.54 ± 5.34	42.76 ± 3.13	76.00 ± 20.42	100.14 ± 12.63*
ALP (U/L)	112.00 ± 9.91	129.60 ± 13.58	122.40 ± 3.23	163.60 ± 36.55	253.00 ± 21.00***	150.80 ± 22.55
GGT (U/L)	0.19 ± 0.14^a^	0.06 ± 0.01^a^	0.05 ± 0.00	0.23 ± 0.18^b^	2.08 ± 1.51^a^	5.65 ± 3.31^a^
TP (g/L)	63.42 ± 1.88	66.46 ± 2.11	65.72 ± 0.38	66.14 ± 2.15	52.08 ± 2.00**	52.88 ± 2.99*
ALB (g/L)	35.96 ± 1.03	37.16 ± 1.16	37.40 ± 0.28	37.84 ± 1.14	29.04 ± 0.90***	28.96 ± 1.33***
TBIL (µmol/L)	1.84 ± 0.47	2.10 ± 0.25	2.30 ± 0.29	2.68 ± 0.10^a^	1.60 ± 0.23	1.10 ± 0.40^c^
CHOL (mmol/L)	1.58 ± 0.15	2.20 ± 0.20	1.68 ± 0.12	1.48 ± 0.19	0.80 ± 0.06*	1.04 ± 0.20
TRIG (mmol/L)	1.46 ± 0.13	1.26 ± 0.09	1.82 ± 0.30	2.12 ± 0.24	0.90 ± 0.21	0.90 ± 0.19
BA (µmol/L)	25.50 ± 6.68	34.74 ± 4.88	22.30 ± 8.69	38.98 ± 8.19	31.32 ± 5.58	69.96 ± 30.57
UREA (mmol/L)	5.44 ± 0.32	4.55 ± 1.14	5.16 ± 0.20	5.34 ± 0.36	5.72 ± 0.35	5.26 ± 0.27
CREAT (µmol/L)	44.78 ± 1.01	45.58 ± 0.85	45.98 ± 1.64	42.34 ± 0.83	47.06 ± 1.64	49.86 ± 2.04
GLUC (mmol/L)	11.60 ± 0.65	10.86 ± 0.63	10.50 ± 1.00	9.44 ± 0.32	13.00 ± 1.89	12.38 ± 1.64
NEFA (mEq/L)	0.56 ± 0.06	0.47 ± 0.04	0.87 ± 0.07	0.78 ± 0.07	0.48 ± 0.07	0.99 ± 0.29
GLOB (g/L)	27.46 ± 0.90	29.30 ± 1.10	28.32 ± 0.21	28.26 ± 1.03	23.04 ± 1.26	23.92 ± 1.74
CK (U/L)	243.22 ± 84.37	492.86 ± 128.98	927.02 ± 298.71	420.04 ± 111.36	1378.24 ± 867.53	3255.10 ± 1358.96*

Data was expressed as mean ± SEM (*n* = 5) unless otherwise stated: ^a^(*n* = 4); ^b^(*n* = 3); ^c^(*n* = 2) due to haemolysis in blood. Note: GGT < 0.1 added as 0.05 in the table for statistical purposes. Control: untreated rats; Na: Sodium; K: Potassium; Mg: Magnesium; Ca: Calcium; PO_4_: Phosphate; PSP: Polysaccharide peptide; Cl: Chloride; CO_2_: Carbon dioxide; AST: Aspartate aminotransferase; ALT: Alanine aminotransferase; ALP: Alkaline phosphatase; GGT: Gamma-glutamyl transpeptidase; TP: Total protein; ALB: Albumin; TBIL: Total bilirubin; CHOL: Cholesterol; TRIG: Triglycerides; BA: Bile acids; UREA: Urea; CREAT: Creatinine; GLUC: Glucose; NEFA: Non-esterified fatty acids: GLOB: Globulin; CK: Creatine kinase. *p < 0.05; **p < 0.01; ***p < 0.001 compared to control (untreated) rats.

There was no significant difference between the untreated control and repeated tamoxifen oral dosing for sodium (Na), magnesium (Mg), phosphate (PO_4_), total bilirubin (TBIL), triglycerides (TRIG), urea (UREA), creatinine (CREAT), glucose (GLUC), non-esterified fatty acids (NEFA), and globulin (GLOB). Although there was a tendency to increase bile acids (BA) with repeated tamoxifen, there was no significant differences compared to untreated control. Repeated tamoxifen oral dosing plus PSP extract tended to reduce BA level compared to untreated control. For cholesterol (CHOL), repeated tamoxifen oral dosing showed comparable levels to untreated control, however, repeated tamoxifen oral dosing plus PSP extract showed reduced cholesterol compared to untreated control (*p* < 0.05).

For potassium (K; *p* < 0.01), chlorine (Cl; *p* < 0.001), creatine kinase (CK; *p* < 0.05), alanine aminotransferase (ALT; *p* < 0.05) and aspartate aminotransferase (AST; *p* < 0.001), there was a significant increase between untreated control and repeated tamoxifen oral dosing levels. Repeated tamoxifen oral dosing plus PSP extract reduced these values compared to untreated control.

For carbon dioxide (CO_2_), there was a significant decrease between untreated control and repeated tamoxifen oral dosing (*p* < 0.05). Repeated tamoxifen oral dosing plus PSP extract increased this to untreated control levels.

For calcium (Ca), there was a significant decrease between untreated control and repeated tamoxifen oral dosing levels (*p* < 0.05). Although there was no significant difference, repeated dose tamoxifen plus extract increased this value slightly.

For TP and ALB, there was a significant decrease between untreated control and repeated tamoxifen oral dosing levels (*p* < 0.05 and *p* < 0.001, respectively). However, there was no significant difference between repeated tamoxifen oral dosing plus PSP extract and repeated tamoxifen oral dosing.

Alkaline phosphatase (ALP) results showed that repeated tamoxifen oral dosing was not significantly different to untreated control (*p* < 0.05). However, there was a significant increase between untreated control and repeated tamoxifen oral dosing plus PSP extract (*p* < 0.001). Although there was an increase in gamma-glutamyl transpeptidase (GGT) with tamoxifen (and a concomitant decrease with tamoxifen plus PSP extract), GGT values were not significantly different between the groups.

### Metabolism of Tamoxifen *In Vitro*

In this study, all microsomal incubation conditions were within the linear range of the reaction rate, and greater than 10% of the substrate was depleted compared to the initial substrate amount (t = 0 min) (Yang et al., 2013). Using substrate depletion constant rates (Obach and Reed-Hagen, 2002), the estimated K_m_ value [substrate concentration at half the maximum velocity (V_max_)] of tamoxifen was 18 μM, indicating that the tamoxifen concentration (5 µM) used in this study was acceptable.

Tamoxifen was stable with microsomal incubation that lacked the NADPH regenerating system indicating that the depletion of tamoxifen in female rat hepatic microsomes was NADPH dependent. Compared with the *in vitro* Cl_int_ of the control (12 ± 4.2 μL/min/mg microsomal proteins), there were no significant differences observed for the *in vitro* Cl_int_ of tamoxifen when co-incubated with different PSP extract concentrations (10, 50, and 100 μg/mL) (Table 4). Consequently, it is unlikely that the PSP extract (between 10 and 100 μg/mL) will significantly inhibit the metabolism of tamoxifen which is pharmacologically mediated *via* CYP activity.

**TABLE 4:** *In vitro* Cl_int_ of tamoxifen (0.5 mM dissolved in 25% acetonitrile) with or without PSP extract in female rat hepatic microsomes.

	Tamoxifen (*n* = 3)	+ PSP extract (10 μg/mL) (*n* = 3)	+ PSP extract (50 μg/mL) (*n* = 3)	+ PSP extract (100 μg/mL) (*n* = 2)
-*k*	0.0060 ± 0.0021	0.0037 ± 0.0022	0.007 ± 0.0025	0.0045 ± 0.0005
*In vitro* t_1/2_ (min)	146.3 ± 46.8	375.4 ± 175.6	123.2 ± 34.2	155.9 ± 17.3
*In vitro* Cl_int_ (µL/min/mg)	12 ± 4.2	7 ± 4	14 ± 5.0	9 ± 1.0

All data is expressed as mean ± SEM of *n* = 3 (10, 50 μg/mL PSP extract), except for 100 μg/mL (*n* = 2) compared to the tamoxifen. *p <* 0.05 was deemed to be statistically significant by using ANOVA (Dunnett’s Multiple Comparison Test).

The disappearance of tamoxifen was also examined in male rat microsomes, and it was found that the clearance rate of tamoxifen was about three-fold faster than the female rat microsome. As in the female microsomes, PSP extract did not appear to alter the depletion of tamoxifen in the male rat microsome (data not shown).

## Discussion

Natural products are increasingly being used as adjunct therapies to support the immune system during anticancer regimens. It is well known that interactions occur between natural products and anticancer drugs, and this can undermine their pharmacological effects (Meijerman et al., 2006). Thus, it is important that patients and clinicians are aware of any potential interactions so that cancer treatment is not compromised.

In this study, the PSP extract interacted with tamoxifen as shown by the increased T_max_ for tamoxifen, without affecting tamoxifen’s bioavailability. One explanation is that the partly digested PSP extract (a highly branched glucan-protein complex) in the gut is slowing gastric emptying time. This lengthens the passage time of tamoxifen in the small intestine and alters the diffusion and interaction of tamoxifen (a lipophilic molecule that is well absorbed from the gastrointestinal tract) with the mucosal surface, and hence its absorption rate (Barsanti et al., 2011). A previous study in rats found that PSK (a polysaccharide similar to PSP) achieved its peak concentration at 0.5–1 h after administration, with the highest concentration observed in the stomach and small intestine and eliminated after 72 h (Ikuzawa et al., 1988). In agreement, our study suggests that the PSP extract was present in the intestinal tract of the rat after 12 h fasting. This delayed emptying of the extract implies that tamoxifen is absorbed more gradually and thus, hindering its acute therapeutic activity (Singh and Malhotra, 2004; Barsanti et al., 2011). However, this may not be clinically relevant as tamoxifen is administered chronically to achieve continued antitumour effects. In rats, tamoxifen acts as a tumoristatic agent—the drug is effective for as long as the drug is present to suppress tumour growth. In humans, long-term tamoxifen therapy increases overall survival. Thus, it is proposed that PSP extract is examined clinically to ascertain whether it sustains tamoxifen’s duration of action (Jordan, 2008).

Tamoxifen can induce biochemical changes (e.g., low levels of DNA adducts in the liver and kidney in rats) and patients are advised to have regular laboratory tests (Li et al., 1997; Farrar and Jacobs, 2020). In this study, PSP extract combined with repeated tamoxifen dosing showed comparable biochemical serum parameter values to the untreated rats, except for ALP, TP, ALB, and CHOL. For ALP, there was a significant increase between control and repeated tamoxifen dosing plus PSP extract (*p* < 0.001). As GGT values were not significantly different between the groups, it is possible that it is not related to hepatobiliary disease but related to bone activity as in a previous study which showed *C. versicolor* stimulated ALP activity in osteoblast cells (Pan et al., 2010).

Furthermore, repeated tamoxifen dosing plus PSP extract significantly decreased CHOL (*p* < 0.05) compared to untreated rats. Tamoxifen has been shown to inhibit cholesterol esterification which is known to promote tumour growth (Poirot et al., 2012). Thus, PSP could aid in inhibiting tumour growth through the lipid pathway.

Another interesting result was a significant decrease in Ca of repeated tamoxifen dosing rats compared to untreated rats (*p* < 0.05). This agrees with a previous rat study (Hemieda, 2007) and in humans which supports the protective effect of tamoxifen against decreased bone mineral release (Resch et al., 1998). PSP extract coadministration did not interfere with this mechanism and the importance of this mechanism requires further clinical investigation.

Overall, the PSP extract is exerting protective effects in combination with tamoxifen. One possible explanation is that β-glucans such as PSP and PSK are known to be orally bioavailable and contribute to the bioactivity of the extract (Ikuzawa et al., 1988; Sullivan et al., 2006; Yu et al., 2013; Saleh et al., 2017). It has been said that molecular weights of no more than 3 kDa are advantageous to ensure passage through the intestinal wall. As this molecular weight was verified for the PSP extract and its capability for intestinal absorption *in vitro*, the extract could be contributing to stabilising biochemical parameters during chronic tamoxifen treatment (Chow and Chu, 2006).

In addition to PSP’s bioavailability, its immunostimulant properties may be due to enhanced colonisation by beneficial microbes in the human gut which would provide health benefits (known as prebiotics). In a previous study, PSP extract was shown to alter human gut microbiota and pH (Yu et al., 2013). Therefore, the time that the PSP extract remains in the gut before its transit may be modifying the intestinal bacteria and modulating the immune system in the rats, thus potentially contributing to favourable biochemical parameters. This is clinically important as chemotherapy has been shown to alter the microbiome which contributes to gut toxicity (Ervin et al., 2020).

This study shows that there were no significant differences between PSP extract-treated rats and untreated rats for all 23 biochemical parameters. This agrees with a previous study examining the acute and subchronic oral toxicity of *C. versicolor* standardised water extract (Hor et al., 2011) thus supporting the extract’s safety profile.

Another important aspect examined in this study was whether PSP interfered with tamoxifen’s metabolism. Tamoxifen is metabolised by a variety of CYP450 enzymes, and the metabolites play an important role in its therapeutic effect (Lien et al., 1991; Mittal et al., 2015). Thus, it is important to preserve this metabolism for effective clinical treatment. In a previous study, PSP competitively inhibited CYP450 enzyme probe substrates metabolism in human liver microsomes *in vitro*. CYP2D6-mediated activity was shown to be minimally inhibited and the relatively high inhibitor constant (K_i_) values for CYP1A2 and CYP3A4 suggested a low potential for PSP to cause natural product-drug interaction related to these CYP isoforms (Yeung and Or, 2012). Another study in humans showed that PSP was safe and was not expected to be associated with significant natural product-drug interactions through CYP3A4 (Nicandro et al., 2007). Using a rat model to investigate CYD 2D-like activity, as well as the potential role of other cytochromes, our results showed that the PSP extract did not interfere with the depletion of tamoxifen and thus, its metabolism, *in vitro*. However, it is well known that species differences exist and this mechanism needs to be investigated clinically.

In the current study, adverse effects were minimal: with tamoxifen, one rat had lost 50 g of weight during extended treatment (with the blood more dense and harder to collect). The limitations of the study included not sampling past the 24 h point, one rat dying at the 10 h sampling point for the single dose tamoxifen plus PSP, not fully elucidating the mechanism of the interaction or immunopotentiation, not measuring the tamoxifen metabolite, unreadable biochemical parameters due to haemolysis and nonidentical weights between the rat groups. Future work could investigate the presence of the polyphenols in *C. versicolor* extracts to ascertain their immunoprotective role (Habtemariam, 2020).

## Conclusion

With increased use of PSP as an adjunct therapy, it is essential that clinicians consider the timing of tamoxifen with PSP so that its clinical actions are not compromised due to slowing its absorption. The study also showed that the coadministration of the PSP extract has the potential to stabilise biochemical changes of long-term tamoxifen therapy which would be welcomed by patients with compromised immune systems. The results also showed that PSP did not inhibit tamoxifen’s metabolism *in vitro* which is important for its clinical effect. Clinical trials will need to be performed to confirm these results.

## Data Availability

The datasets generated and analysed during this study are available from the corresponding authors on reasonable request.
